# A Macrophage‐Derived 7‐Gene Signature Predicts Prognosis and Therapeutic Response in Hepatocellular Carcinoma

**DOI:** 10.1002/iub.70079

**Published:** 2025-12-07

**Authors:** Xiaomin Li, Zhilong Li, Jinfang Zhai, Binbin Zou

**Affiliations:** ^1^ General Surgery Department, Shanxi Bethune Hospital Third Hospital of Shanxi Medical University Taiyuan China; ^2^ Thoracic Surgery Department, Shanxi Cancer Hospital/Shanxi Hospital Affiliated to Cancer Hospital Chinese Academy of Medical Sciences/Cancer Hospital Affiliated to Shanxi Medical University Taiyuan China; ^3^ Respiratory Department, Shanxi Cancer Hospital/Shanxi Hospital Affiliated to Cancer Hospital Chinese Academy of Medical Sciences/Cancer Hospital Affiliated to Shanxi Medical University Taiyuan China; ^4^ Rheumatology Department, Shanxi Bethune Hospital Third Hospital of Shanxi Medical University Taiyuan China; ^5^ Academy of Medical Sciences Shanxi Medical University Taiyuan China

**Keywords:** EGFR tyrosine kinase inhibitor resistance, hepatocellular carcinoma, LASSO, macrophages, prognostic signature, single‐cell RNA sequencing, tumor microenvironment

## Abstract

This study aimed to identify a novel prognostic signature derived from an EGFR Tyrosine kinase inhibitors (TKI‐resistant) macrophage subpopulation and to evaluate its clinical and therapeutic relevance in HCC. We utilized single‐cell RNA sequencing data from HCC patients. An EGFR‐TKI resistance score was calculated across all cell types. Macrophages, which exhibited the highest resistance score, were sub‐clustered to identify the most resistant subpopulation. Marker genes from this sub‐cluster were intersected with differentially expressed genes (DEGs) from the TCGA‐LIHC cohort. A robust prognostic model was constructed. The model's performance was rigorously validated, and the signature was further characterized through multi‐omics analysis and its correlation with immune checkpoint blockade (ICB) response and drug sensitivity. scRNA‐seq analysis unequivocally identified macrophages as possessing the highest EGFR‐TKI resistance score. We identified seven key prognostic genes: *SLC41A3*, *DCAF13*, *PPM1G*, *NDC80*, *FAM83D*, *FUCA2*, and *UQCRH*. A risk model built on these seven genes effectively stratified patients into high‐ and low‐risk groups with significantly different overall survival (OS) in the TCGA cohort, a finding successfully validated in the independent GSE76427 cohort. A clinical nomogram integrating the risk score demonstrated excellent predictive accuracy, with AUC values for 1‐, 3‐, and 5‐year OS of 0.816, 0.781, and 0.799, respectively. The low‐risk group was associated with a favorable immune‐infiltrated phenotype and was predicted to be more sensitive to immunotherapy. Conversely, the high‐risk group exhibited distinct genomic features and was predicted to be more sensitive to specific targeted agents, including Navitoclax and Sorafenib. We identified and validated a novel 7‐gene prognostic signature derived from a subpopulation of EGFR‐TKI‐resistant macrophages. This signature accurately predicts patient survival, offers insights into the molecular mechanisms of therapy resistance in HCC, and provides a promising tool for improved patient stratification and the development of personalized treatment strategies.

## 1 Introduction

Primary liver cancer (PLC) is the sixth most commonly diagnosed malignancy and the third leading cause of cancer‐related death globally, with ~906,000 new cases and 830,000 deaths annually [1]. Hepatocellular carcinoma (HCC) constitutes 75%–85% of all PLC cases [2]. Due to the absence of obvious clinical symptoms in the early stages, most patients are diagnosed at an advanced stage, leading to a poor prognosis [3]. For patients diagnosed early, surgical resection and liver transplantation remain the primary curative treatments. However, a majority of patients have already lost the opportunity for curative surgery upon diagnosis [4].

Systemic therapy is the standard of care for patients with advanced HCC. Tyrosine kinase inhibitors (TKIs), such as sorafenib and lenvatinib, have long been the cornerstone of first‐line treatment [5]. Despite their initial efficacy, acquired resistance to TKIs frequently develops, limiting long‐term patient benefit. In parallel, immunotherapy, particularly immune checkpoint inhibitors (ICIs) targeting the PD‐1/PD‐L1 axis, has emerged as a powerful therapeutic strategy [6]. However, the response rates to ICIs as monotherapy are modest, with only a subset of patients experiencing durable clinical benefit [7]. This highlights the urgent need to understand the mechanisms underlying therapeutic resistance and to develop robust biomarkers for patient stratification and treatment selection.

The tumor microenvironment (TME) is a complex and dynamic ecosystem of cancer cells, stromal cells, immune cells, and extracellular matrix components, which profoundly influences tumor progression, metastasis, and response to therapy [8]. In recent years, single‐cell RNA sequencing (scRNA‐seq) has revolutionized our ability to dissect the heterogeneity of the TME at an unprecedented resolution [9]. This technology allows for the detailed characterization of diverse cell populations and their intricate interactions, providing critical insights into the mechanisms of tumorigenesis and drug resistance [10].

Within the TME, cytotoxic T lymphocytes (CTLs) are the primary effectors of anti‐tumor immunity, yet their function is often suppressed in established tumors. This state of T‐cell dysfunction, or “exhaustion,” is a major barrier to effective immunotherapy [11]. Tumor‐associated macrophages (TAMs) are key architects of this immunosuppressive landscape. TAMs can directly inhibit T‐cell proliferation and function through the expression of checkpoint ligands like PD‐L1 and the secretion of immunosuppressive cytokines such as IL‐10 and TGF‐β [12, 13]. Understanding the cellular drivers of therapy resistance is therefore paramount.

In this study, we leveraged scRNA‐seq data to investigate the cellular basis of EGFR‐TKI resistance in HCC. We hypothesized that a specific cell type within the TME is a primary contributor to resistance. We identified macrophages as having the highest EGFR‐TKI resistance signature, isolated a key resistant subpopulation, and developed a novel 7‐gene prognostic model based on its marker genes. We thoroughly validated this model using data from The Cancer Genome Atlas (TCGA) and explored its correlation with the genomic landscape, immune features, and drug sensitivity, aiming to provide a new tool for prognostic assessment and personalized therapy in HCC.

## 2 Materials and Methods

### 2.1 Single‐Cell RNA Sequencing Data Processing

All single‐cell RNA‐sequencing (scRNA‐seq) data processing was performed in R (v4.4.3) using the Seurat package (v5.0). Raw gene expression matrices were loaded, and Seurat objects were created. Initial quality control (QC) was applied to filter out low‐quality cells based on thresholds for the number of unique genes detected and the percentage of mitochondrial reads. Post‐filtering, data was normalized using the NormalizeData function (LogNormalize method) and scaled using ScaleData, while optionally regressing out unwanted variation from mitochondrial content. The top 2000 highly variable genes (HVGs) were identified using FindVariableFeatures (“vst” method) and used for linear dimensionality reduction via Principal Component Analysis (PCA) with RunPCA. A K‐nearest neighbor (KNN) graph was built using FindNeighbors, and cell clusters were identified using the Louvain algorithm with FindClusters. Clusters were visualized using Uniform Manifold Approximation and Projection (UMAP). We subsequently identified distinct cell clusters and meticulously annotated them using established marker genes for major lineages: T cells (*CD3D*, *CD3E*), B cells (*CD79A*, *IGHG1*), endothelial cells (*PECAM1*, *VWF*), myeloid cells (*CD1C*), and epithelial/hepatocyte cells (*EPCAM*, *HNF4A*). Cluster‐specific marker genes were identified using the FindAllMarkers function (Wilcoxon Rank Sum test).

### 2.2 EGFR Tyrosine Kinase Inhibitor Resistance Score Calculation

To quantify the EGFR tyrosine kinase inhibitor resistance phenotype at the single‐cell level, we calculated a resistance score for each cell. A curated gene set associated with EGFR‐TKI resistance was obtained from the Molecular Signatures Database (MSigDB). Four different algorithms—ssGSEA, AUCell, UCell, and singscore—implemented via the irGSEA R package, were employed to compute the enrichment score for this gene set in each cell. They rely on distinct and complementary computational principles to quantify gene set activity within a single sample. The cell type with the consistently highest score across all four algorithms was selected for downstream analysis.

### 2.3 Identification of Resistance‐Associated Macrophage Marker Genes

The macrophage population was isolated from the dataset and re‐clustered to identify distinct subpopulations. The EGFR tyrosine kinase inhibitor resistance score was recalculated for each macrophage sub‐cluster. Marker genes for the sub‐cluster with the highest resistance score were identified using the FindMarkers function in the Seurat R package, with a log‐fold change threshold > 0.25 and an adjusted *p* value < 0.05.

### 2.4 TCGA Data Acquisition and Identification of DEGs

Differentially expressed genes (DEGs) between HCC tumor tissues and adjacent normal tissues were identified using the GEPIA2 web server [14]. The marker genes from the resistant macrophage sub‐cluster were then intersected with the TCGA‐LIHC DEGs to identify a core set of candidate prognostic genes.

### 2.5 Construction and Validation of a Prognostic Gene Signature

The TCGA‐LIHC cohort was randomly divided into a training set and a validation set. In the training set, Least Absolute Shrinkage and Selection Operator (LASSO) Cox regression analysis was performed on the intersected candidate genes to select the most prognostically relevant genes and to prevent overfitting. A risk score was calculated for each patient using the following formula:

$$\text{RiskScore}=\sum_{i=n}^{n}\left({\text{Coef}}_{i}\times {\text{Expr}}_{i}\right)$$

where *Coefᵢ* is the LASSO regression coefficient for gene *i*, and *Exprᵢ* is its expression level.

Patients in the training, validation, and entire TCGA cohorts were stratified into high‐risk and low‐risk groups based on the median risk score. Kaplan–Meier survival analysis with the log‐rank test was used to compare the overall survival (OS) between the two groups. Time‐dependent Receiver Operating Characteristic (ROC) curves and the Area Under the Curve (AUC) were used to evaluate the predictive performance of the model at 1, 3, and 5 years.

### 2.6 Independent Prognostic Analysis and Nomogram Construction

Univariate and multivariate Cox regression analyses were performed to assess whether the risk score was an independent prognostic factor when considered alongside other clinical variables such as age, gender, grade, and tumor stage. Subsequently, a nomogram prognostic model was constructed using the rms R package, incorporating all independent prognostic factors identified in the multivariate analysis. The predictive accuracy of the nomogram was evaluated by plotting ROC curves and calculating the AUC for 1‐, 3‐, and 5‐year survival.

### 2.7 Multi‐Omics and Functional Analysis of the Signature

We analyzed the expression of the seven signature genes across different cell types in the scRNA‐seq data and their differential expression in the TCGA‐LIHC cohort. We also evaluated their individual prognostic value using Kaplan–Meier analysis. The correlation between the seven genes was calculated using Pearson correlation. Somatic mutation data (MAF files) for the TCGA‐LIHC cohort were analyzed to compare the Tumor Mutational Burden (TMB) between high‐ and low‐risk groups and to visualize the mutation landscape of the signature genes. Copy Number Variation (CNV) and DNA methylation data were also analyzed to investigate their association with the expression and prognostic significance of the signature genes.

### 2.8 Immune Response and Drug Sensitivity Prediction

The Tumor Immune Dysfunction and Exclusion (TIDE) algorithm [15] was used to predict the potential response to immune checkpoint blockade therapy. TIDE scores, dysfunction scores, and exclusion scores were compared between the high‐ and low‐risk groups. To explore potential therapeutic strategies, we analyzed the correlation between the risk score and drug sensitivity (IC50 values) using two large‐scale pharmacogenomic databases: the Genomics of Drug Sensitivity in Cancer (GDSC) [16] and the Cancer Therapeutics Response Portal (CTRP) [17] from the GSCA database.

The oncoPredict R package was used to calculate the differential drug sensitivity between high‐ and low‐risk groups. Additionally, public immunotherapy scRNA‐seq datasets (GSE206325 and Liu_HCC from the scICB database) were analyzed to examine the differential expression of model genes between responder and non‐responder patient groups.

### 2.9 Statistical Analysis

All statistical analyses were performed using R software. The Wilcoxon rank‐sum test was used for comparisons between two groups. The log‐rank test was used for Kaplan–Meier survival analysis. A *p* value < 0.05 was considered statistically significant.

## 3 Results

### 3.1 Macrophages Exhibit the Highest EGFR‐TKI Resistance Score in the HCC Microenvironment

**FIGURE 1:**
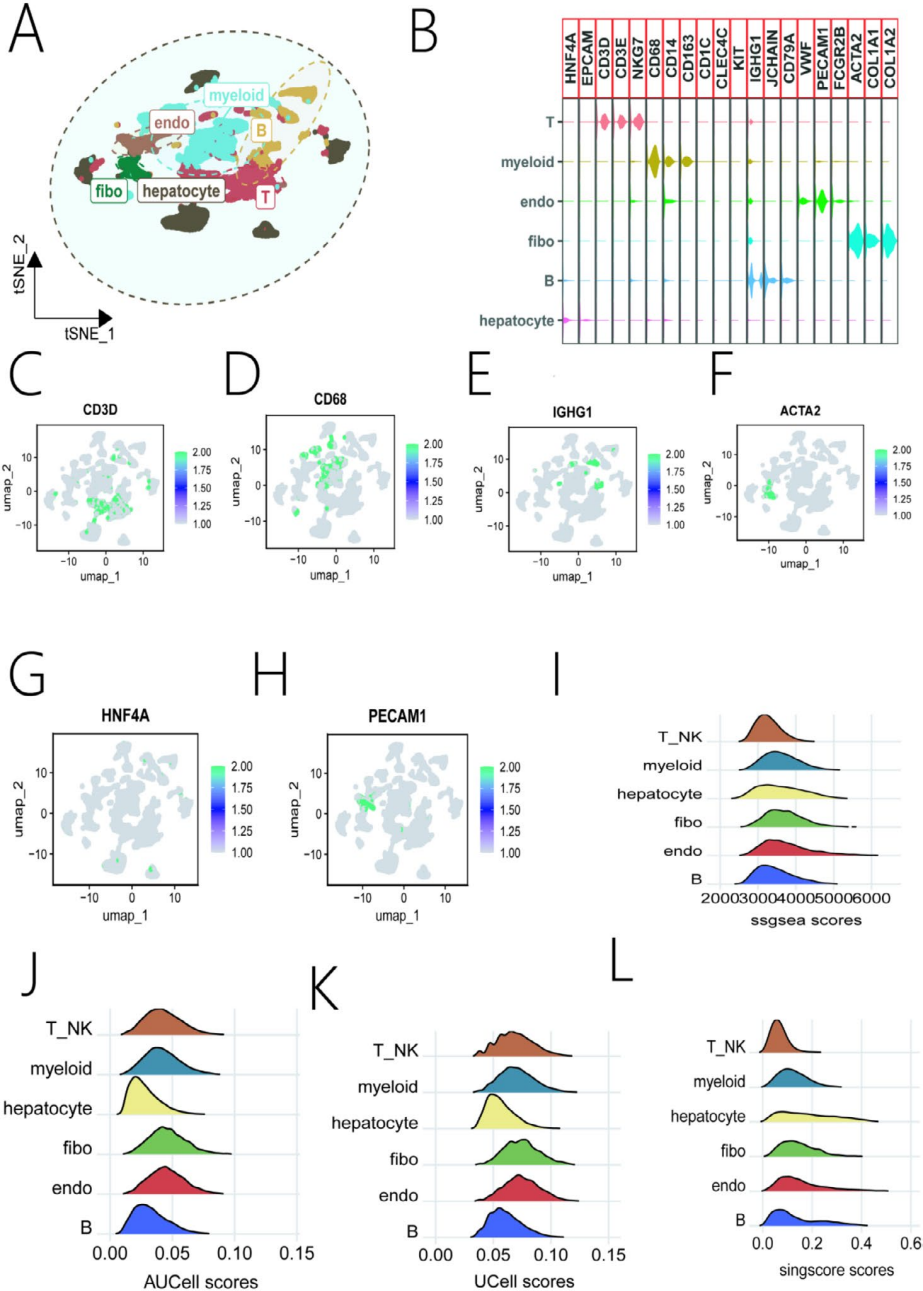
Single‐cell RNA sequencing analysis of the HCC microenvironment and EGFR‐TKI resistance. (A) UMAP plot showing the distribution of six different cell types. (B) Dot plot illustrating the expression levels of marker genes across cell types. (C–H) Feature plots showing the expression of representative marker genes for each cell type. (I‐L) Showing the resistance gene set scores for each cell type, calculated using (I) ssGSEA, (J) AUCell, (K) UCell, and (L) singscore algorithms.

After quality control of the scRNA‐seq data from the GSE149614 dataset, a total of 71,915 cells were retained for analysis. Unsupervised clustering identified six major cell types: T cells, B cells, endothelial cells, myeloid cells, and epithelial/hepatocyte cells (Figure 1A). The identity of these clusters was confirmed by the expression of canonical marker genes (Figure 1B–H). To investigate the cellular origins of therapy resistance, we calculated an EGFR tyrosine kinase inhibitor resistance score for each cell using four independent algorithms. All four methods consistently revealed that macrophages had the highest resistance score among all cell types (Figure 1I–L), suggesting that macrophages are a key contributor to EGFR‐TKI resistance in the HCC microenvironment.

### 3.2 Identification of a Resistance‐Associated Macrophage Sub‐Cluster and Core Genes

**FIGURE 2:**
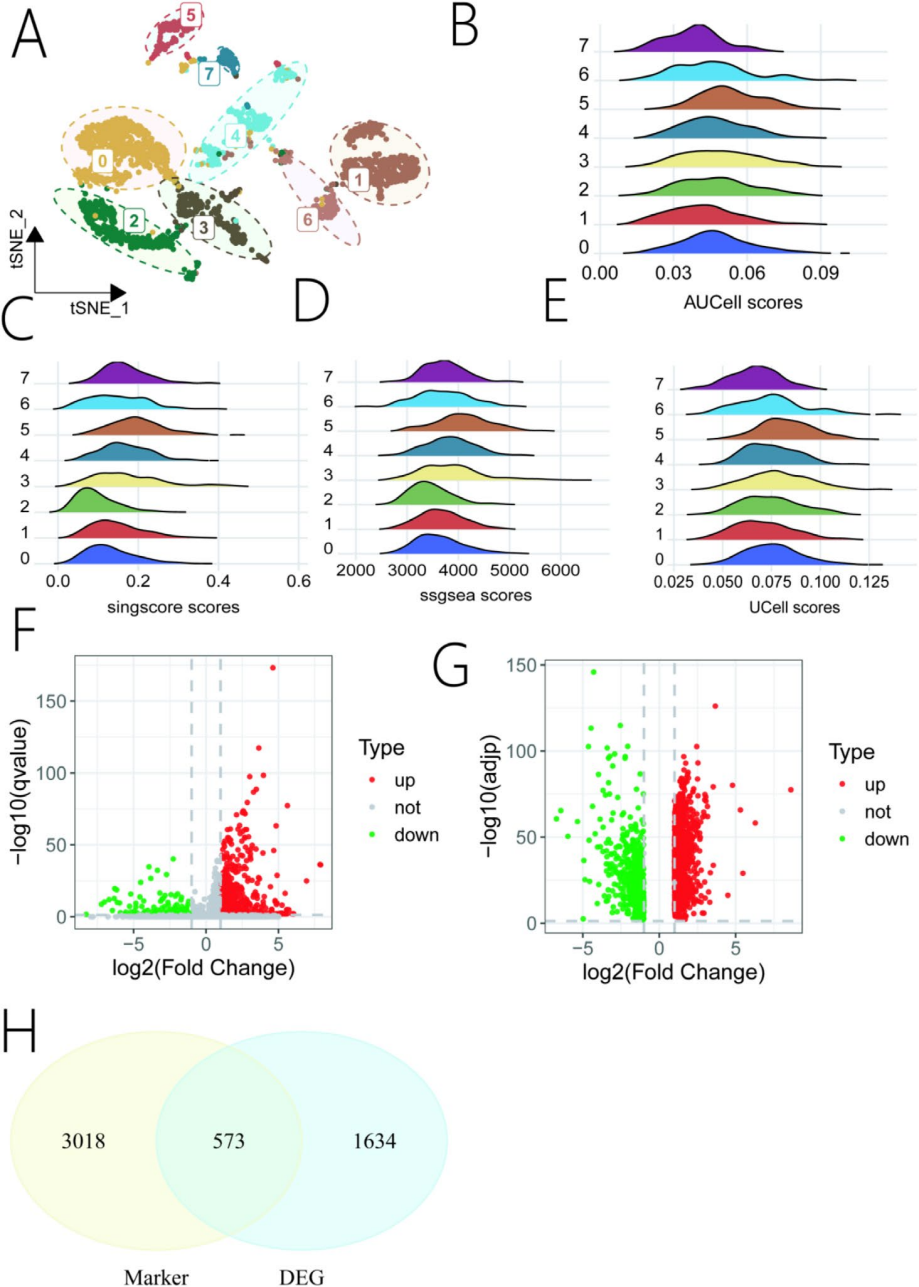
Analysis of EGFR‐TKI resistance in Macrophage subpopulations. (A) UMAP plot showing the eight distinct Macrophage sub‐clusters. (B–E) Gene set scores for each Macrophage sub‐cluster based on (B) AUCell, (C) singscore, (D) ssGSEA, and (E) UCell algorithms. (F) Volcano plot showing the marker genes of the Macrophage_5 sub‐cluster relative to other sub‐clusters. (G) Volcano plot of differentially expressed genes (DEGs) between tumor and normal tissues from the GEPIA2 database. (H) Venn diagram showing the intersection of Macrophage_5 marker genes and TCGA‐LIHC DEGs.

To further explore the heterogeneity within the macrophage population, we extracted and re‐clustered the macrophage cells, which yielded eight distinct sub‐clusters (Figure 2A). We then recalculated the EGFR‐TKI resistance score for each macrophage sub‐cluster. Again, all four algorithms consistently identified the Macrophage_5 sub‐cluster as having the highest resistance score (Figure 2B–E). We identified 3591 marker genes that were significantly different in expression in the Macrophage_5 sub‐cluster compared with other macrophage sub‐clusters (Figure 2F). Concurrently, we identified 2227 DEGs between HCC and normal tissues from the TCGA‐LIHC dataset using GEPIA2 (Figure 2G). By intersecting the Macrophage_5 marker genes with the TCGA DEGs, we obtained a final set of 573 common genes for subsequent prognostic model construction (Figure 2H).

### 3.3 Construction and Validation of a 7‐Gene Prognostic Signature

**FIGURE 3:**
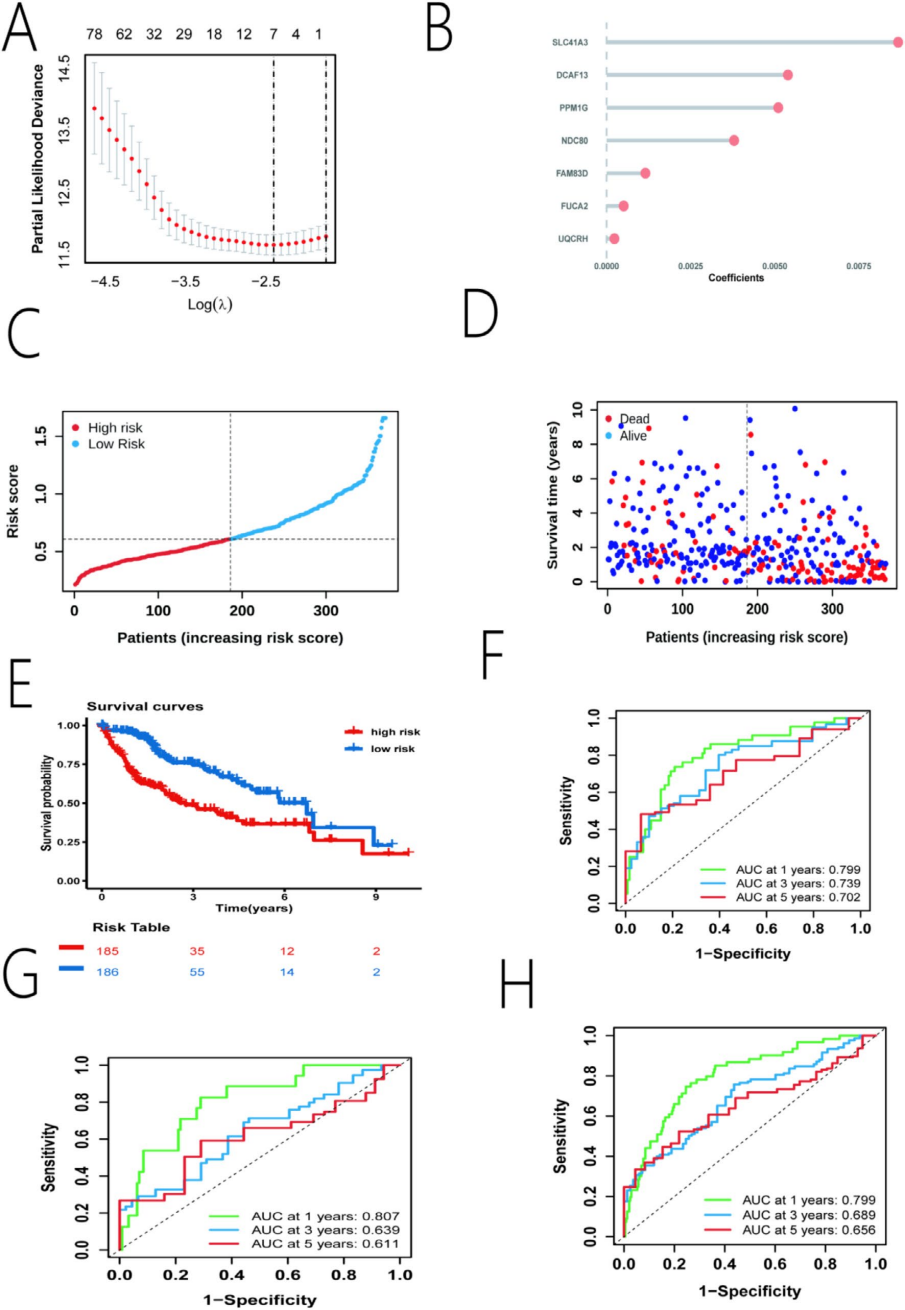
Construction and validation of the prognostic model. (A) LASSO regression analysis showing the gene coefficient profiles as a function of the log(λ) value. (B) Regression coefficients of the seven selected genes in the risk model. (C, D) Distribution of risk scores and patient survival status in the training cohort. (E) Kaplan–Meier survival curves for high‐ and low‐risk groups in the entire TCGA cohort. (F–H) Time‐dependent ROC curves for 1‐, 3‐, and 5‐year OS prediction in the (F) training set, (G) validation set, and (H) entire cohort.

Using LASSO Cox regression analysis on the 573 candidate genes in the TCGA‐LIHC data, we identified a robust 7‐gene signature for predicting overall survival. The final model included *SLC41A3*, *DCAF13*, *PPM1G*, *NDC80*, *FAM83D*, *FUCA2*, and *UQCRH* (Figure 3A,B). A risk score was calculated for each patient. Patients were then divided into high‐risk and low‐risk groups based on the median risk score. The distribution of risk scores and patient survival status in the training set showed that patients with higher risk scores had poorer outcomes (Figure 3C,D). Kaplan–Meier survival analysis demonstrated that patients in the high‐risk group had significantly shorter overall survival than those in the low‐risk group (Figure 3E). The predictive performance of the model was assessed using time‐dependent ROC curves. In the training set, the AUC values for predicting 1‐, 3‐, and 5‐year OS were 0.799, 0.739, and 0.702, respectively (Figure 3F). The model's robustness was confirmed in the validation set (AUCs: 0.807, 0.639, 0.666) and the entire TCGA cohort (AUCs: 0.799, 0.689, 0.656) (Figure 3G,H), indicating its strong and stable prognostic capability.

### 3.4 Risk Signature Model Optimization and External Validation

**FIGURE 4:**
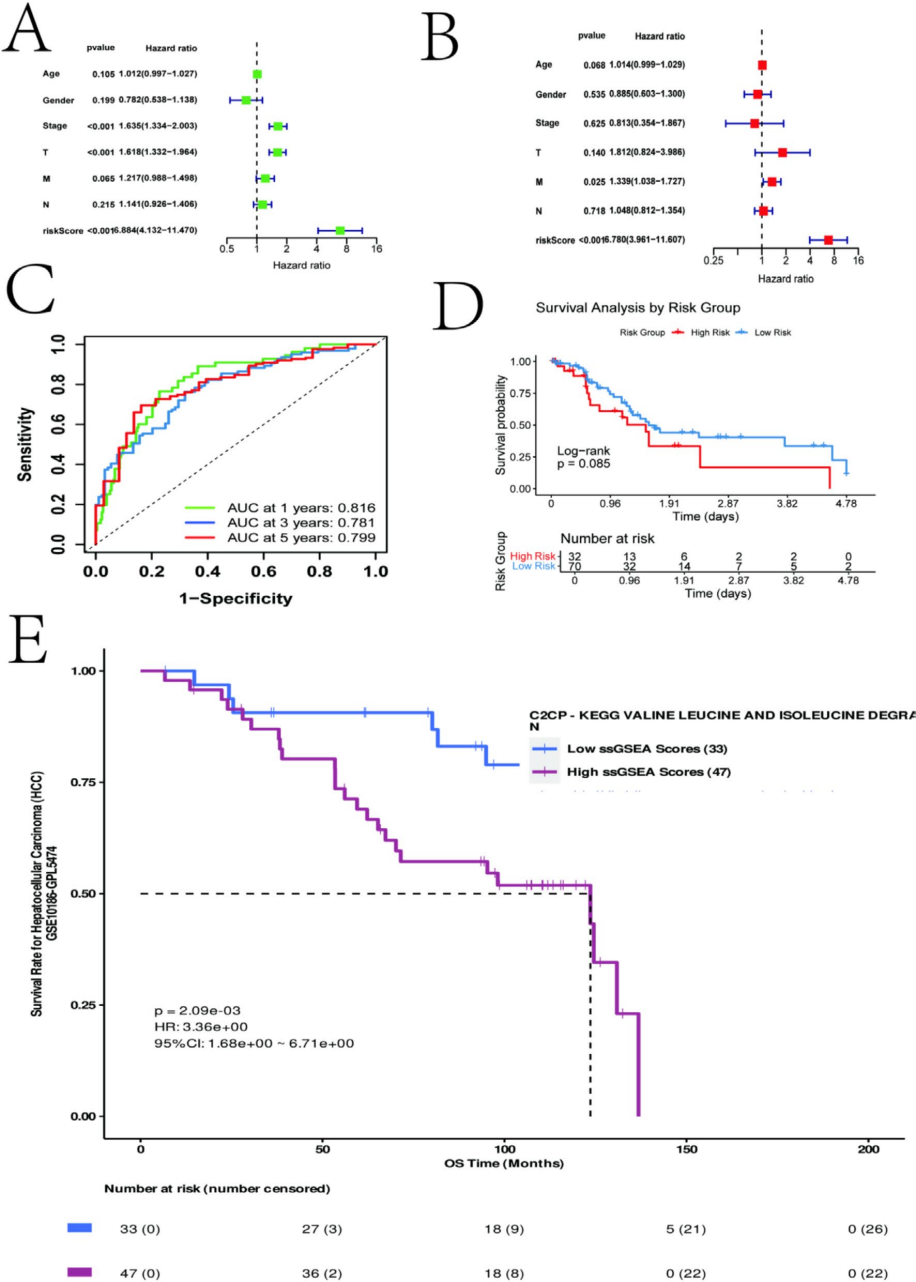
Risk signature model optimization and other data validation. (A) Univariate analysis, (B) Multivariate analysis, (C) Column charts 1, 3, 5 AUC curves, (D) GSE76427 high/low risk score survival curves, (E) GSE10186 BCAA GSVA high/low score survival curves.

Through univariate and multivariate Cox regression analyses of the risk score and standard clinical characteristics, we confirmed that the risk score was an independent prognostic factor for OS (Figure 4A,B). By constructing a nomogram that integrated the risk score with other independent clinical factors, we observed improved predictive performance, with AUC values of 0.816, 0.781, and 0.799 for 1‐, 3‐, and 5‐year survival, respectively (Figure 4C). To assess the generalizability of our signature, we performed validation in the independent GSE76427 dataset. Consistent with our findings in the TCGA cohort, patients in the high‐risk group exhibited worse prognosis (Figure 4D). Furthermore, we investigated the prognostic value of a BCAA‐related gene set in the GSE10186 dataset, finding that a low BCAA GSVA score was associated with significantly better outcomes (Figure 4E), with similar trends observed in GSE27150 (Figure S1).

### 3.5 Expression and Prognostic Value of the Signature Genes

**FIGURE 5:**
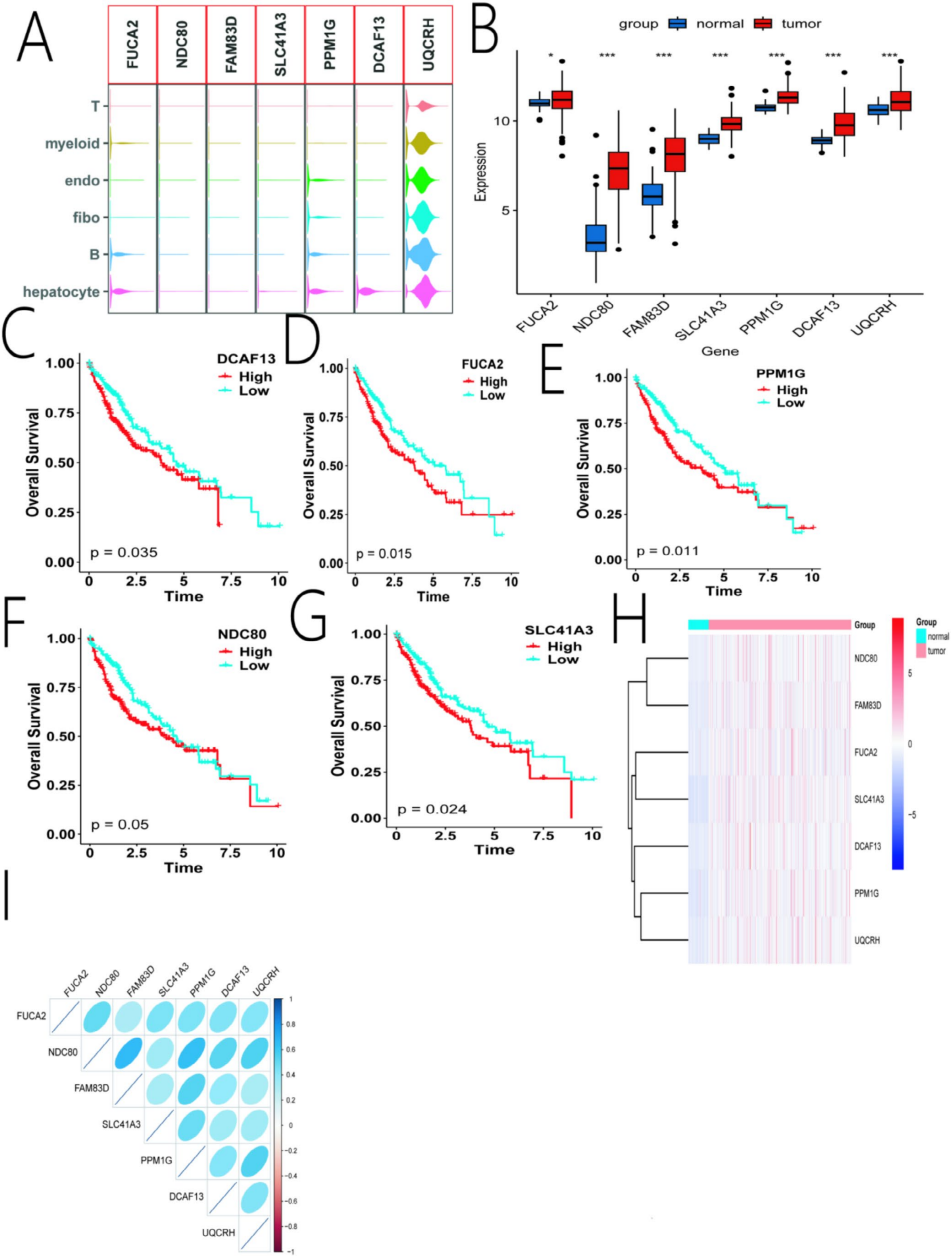
Expression and clinical relevance of the prognostic signature genes in the TCGA‐LIHC dataset. (A) Violin plot showing the expression levels of the 7 signature genes across different cell types in the scRNA‐seq data. (B) Boxplot comparing the expression of the 7 genes in tumor vs. normal tissues. (C–G) Kaplan–Meier survival analysis for individual signature genes. (H) Heatmap showing the differential expression of the 7 genes in tumor versus normal tissues. (I) Correlation matrix plot displaying the Pearson correlation coefficients between the 7 genes (*p* < 0.05; **p < 0.01; ***p < 0.001).

Analysis of the seven signature genes in the scRNA‐seq data showed that they were highly expressed in hepatocytes/epithelial cells (Figure 5A). In the TCGA‐LIHC bulk RNA‐seq data, all seven genes were significantly upregulated in tumor tissues compared with adjacent normal tissues (Figure 5B,H). Furthermore, survival analysis for each individual gene revealed that high expression of these genes was significantly associated with worse overall survival (Figure 5C–G). Correlation analysis showed a strong positive correlation among the expression levels of the seven genes, particularly between *NDC80* and *UQCRH*, *PPM1G*, and *FAM83D* (Figure 5I), suggesting they may be co‐regulated or function in related pathways.

### 3.6 Genomic Alterations, CNV, and DNA Methylation Analysis of Signature Genes

**FIGURE 6:**
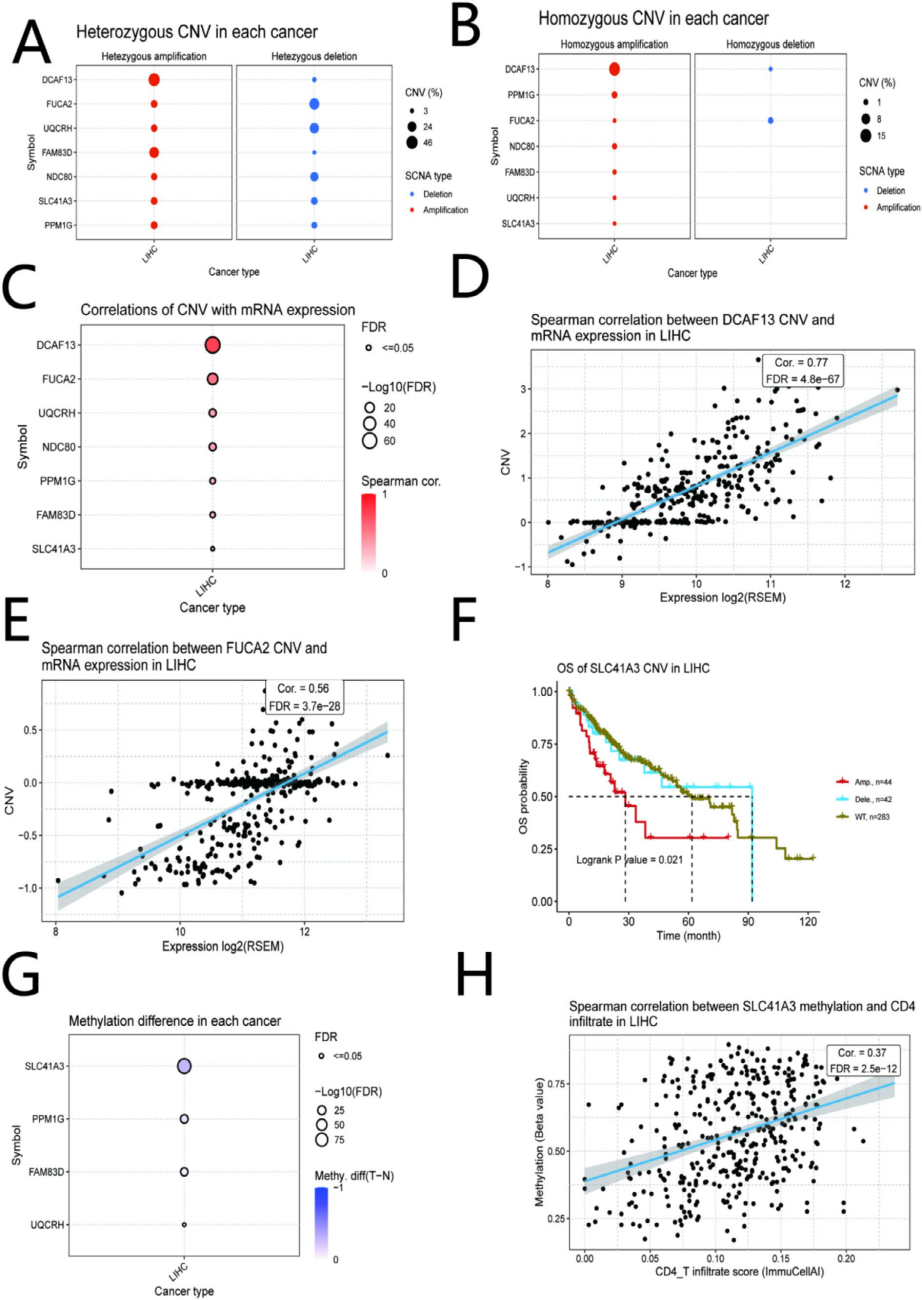
CNV and DNA methylation analysis of the signature genes in TCGA‐LIHC. (A) Expression of the seven genes across heterozygous CNV states. (B) Expression across homozygous CNV states. (C) Correlation between CNV and mRNA expression for the seven genes. (D, E) Scatter plots showing the correlation for (D) *DCAF13* and (E) *FUCA2*. (F) Kaplan–Meier analysis based on *SLC41A3* CNV status. (G) DNA methylation levels of four signature genes in tumor vs. normal tissues. (H) Correlation between *SLC41A3* methylation and CD4+ T cell infiltration.

We next investigated the relationship between the risk score and the genomic landscape in the TCGA‐LIHC cohort. The mutation frequency of the 7 signature genes in LIHC was very low, with all genes mutated in < 5% of cases (Figure S2). We further analyzed the copy number variation (CNV) and DNA methylation status of the seven signature genes. Both heterozygous and homozygous CNV analyses showed significant amplification for *DCAF13* and *FAM83D* and deletion for *FUCA2*, *UQCRH*, and *NDC80* in tumor samples (Figure 6A,B). The mRNA expression levels of all seven genes were positively correlated with their CNV levels (Figure 6C), with the strongest correlations observed for *DCAF13* (*r* = 0.77) and *FUCA2* (*r* = 0.56) (Figure 6D,E). Notably, patients with *SLC41A3* amplification had the worst overall survival (Figure 6F). In terms of DNA methylation, *SLC41A3*, *PPM1G*, *FAM83D*, and *UQCRH* all showed significantly lower methylation levels in tumor tissues compared with normal tissues, with the most significant decrease observed for *SLC41A3* (Figure 6G). Furthermore, the methylation level of *SLC41A3* was positively correlated with CD4+ T cell infiltration (*r* = 0.37), linking its epigenetic regulation to the immune microenvironment (Figure 6H).

### 3.7 Association of the Prognostic Model With the Immune Microenvironment

**FIGURE 7:**
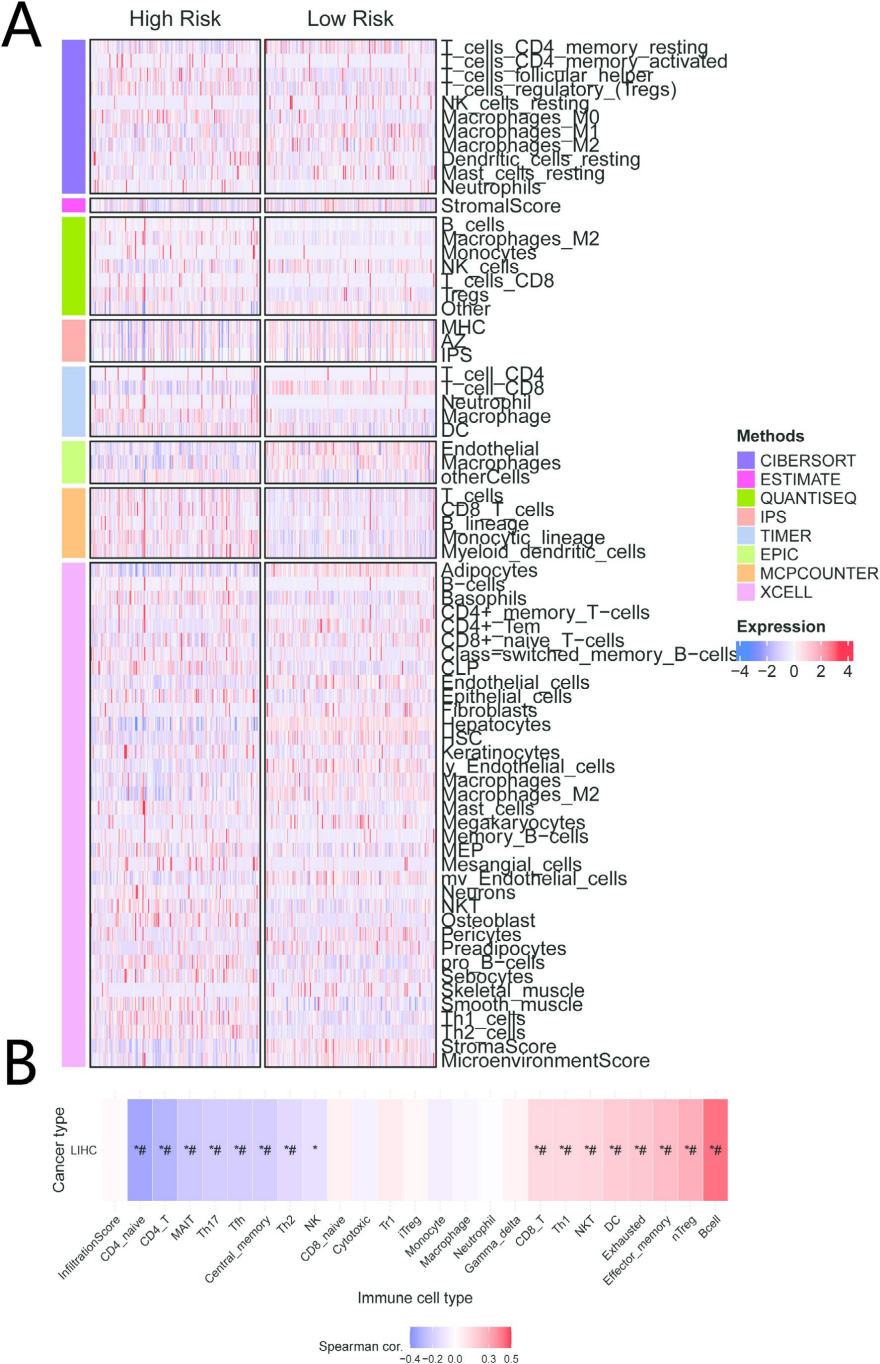
Analysis of immune cell infiltration characteristics between high‐risk and low‐risk groups. (A) The heat map illustrates the distribution of immune and stromal cells between high‐risk and low‐risk groups using seven immunoinfiltration estimation methods (CIBERSORT, ESTIMATE, QUANTISEQ, IPS, TIMER, EPIC, MCPcounter, and xCell). Each row represents an immune or stromal cell type, with each column corresponding to a sample. Red indicates relatively high expression, while blue denotes relatively low expression. (B) Spearman correlation analysis chart demonstrates the correlation between immune cell infiltration levels and risk scores (based on LIHC cohort). Red dots indicate immune cells positively correlated with high risk, while blue dots show negative correlations. The asterisk (*) signifies statistically significant results (*p < 0.05).

To investigate the relationship between risk groups and the immune microenvironment, we employed eight algorithms (CIBERSORT, ESTIMATE, MCPcounter, EPIC, IPS, quantiSEQ, xCell) to evaluate immune cell composition in TCGA‐LIHC. Our analysis revealed that low‐risk groups exhibited higher enrichment of various immune cells, particularly cytotoxic T‐cells (e.g., T‐cell CD8+, T‐cell CD4+ Memory Resting) (Figures 7A, S3, and S4). Correlation analysis confirmed that the risk score was negatively associated with the infiltration of multiple anti‐tumor immune cell types (Figure 7B) in the GSCA database. These findings suggest that the favorable prognosis of the low‐risk group may be driven by a more robust anti‐tumor immune response.

### 3.8 Correlation of the Risk Signature With Drug Sensitivity

**FIGURE 8:**
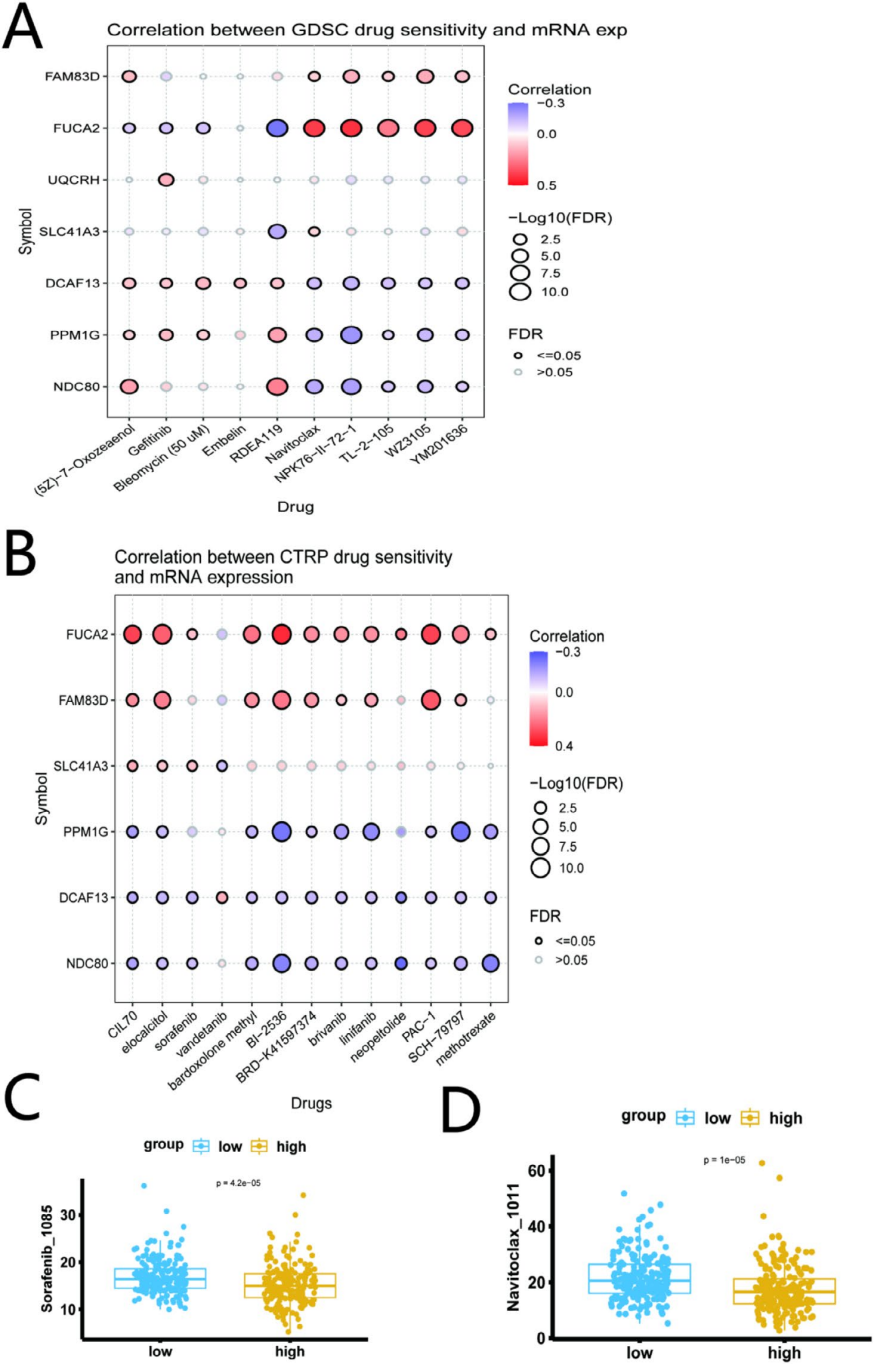
Correlation between signature gene expression and drug sensitivity. (A) Correlation analysis based on the GDSC database. (B) Correlation analysis based on the CTRP database. In both plots, red indicates a positive correlation (sensitivity), and blue indicates a negative correlation (resistance). The size of the circle represents statistical significance (FDR). (C,D) The sensitivity to Sorafenib and Navitoclax in the low and high risk group.

To identify potential therapeutic agents, we analyzed the correlation between the mRNA expression of the seven signature genes and drug sensitivity using the GDSC and CTRP databases from the GSCA database. In the GDSC, high expression of *FUCA2* was associated with sensitivity to multiple drugs, including the MEK inhibitor PD‐0325901, the EGFR inhibitor Erlotinib, and the BCL‐2 inhibitor Navitoclax. Conversely, high expression of *PPM1G* and *FAM83D* was associated with resistance to several compounds (Figure 8A). A similar pattern was observed in the CTRP database, where *FUCA2* expression correlated positively with sensitivity to numerous agents like etoposide and bortezomib, while *PPM1G* and *NDC80* expression correlated with resistance (Figure 8B). Importantly, when analyzing sensitivity based on the integrated risk score, we found that the high‐risk group was significantly more sensitive to Navitoclax and the TKI Sorafenib (Figure 8C,D). These results suggest that *FUCA2* could be a marker for drug sensitivity, whereas *PPM1G* and *NDC80* might contribute to multi‐drug resistance.

### 3.9 Correlation of the Risk Signature With Immune Response

**FIGURE 9:**
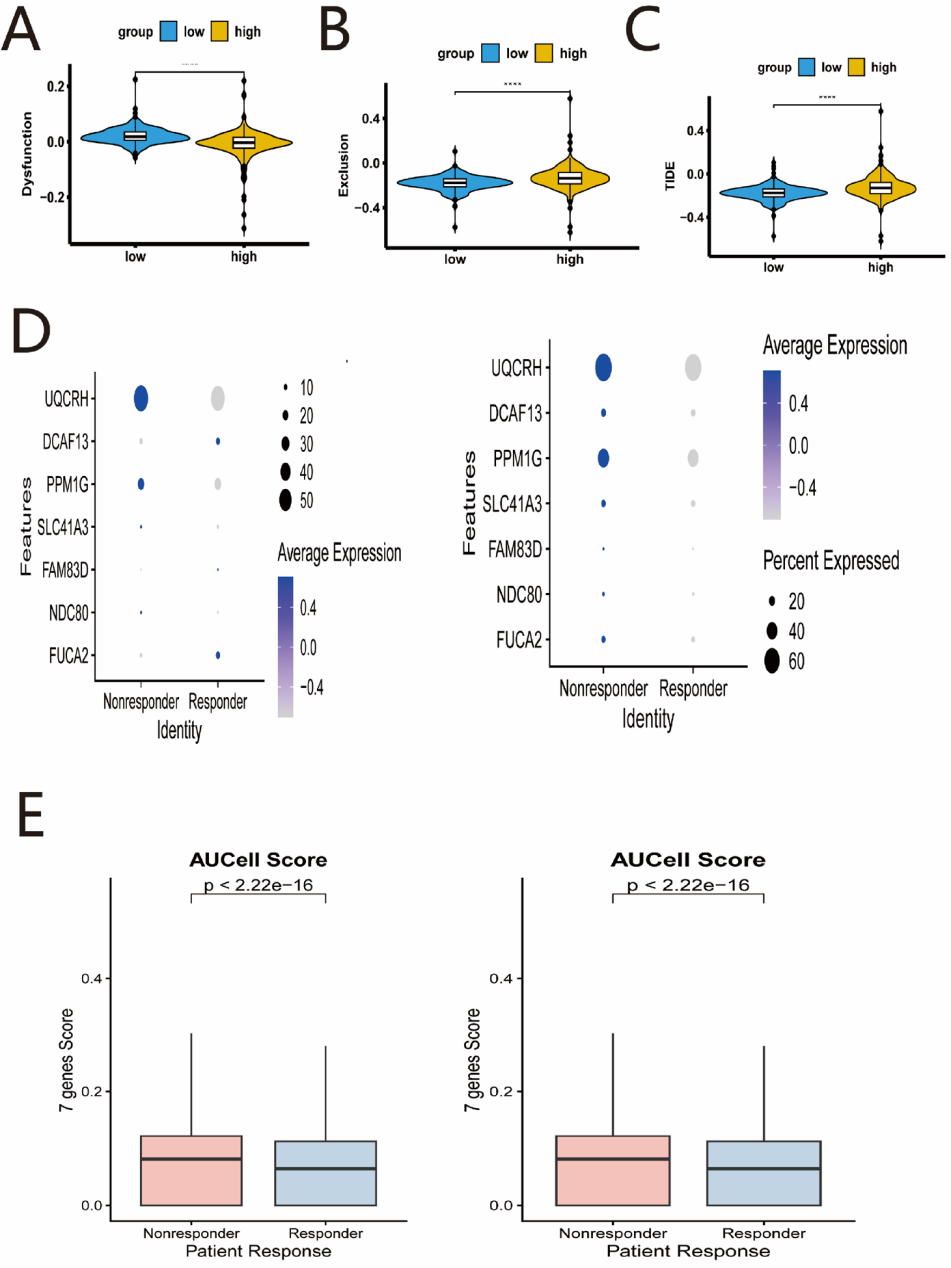
Immunotherapy response analysis associated with the risk score. Comparison of (A) TIDE score, (B) T‐cell dysfunction score, and (C) T‐cell exclusion score between risk groups. (D) Gene expression in response and non‐response groups to immunotherapy in the GSE206325 and Liu_HCC datasets. (E) the seven genes score in response and non‐response groups to immunotherapy in the GSE206325 and Liu_HCC datasets.

To evaluate the potential for immunotherapy response, we used the TIDE algorithm. The high‐risk group had significantly higher TIDE and T‐cell exclusion scores and a lower T‐cell dysfunction score compared with the low‐risk group (Figure 9A–C). This suggests that the poor prognosis in the high‐risk group might be driven by an immune‐excluded phenotype, while the low‐risk group may be more likely to benefit from immune checkpoint inhibitors. In two independent immunotherapy single‐cell datasets, GSE206325 and Liu_HCC from the scICB database, the expression of *UQCRH* and *PPM1G* was significantly lower in the responder group compared with the non‐responder group (Figure 9D), and the seven genes score was significantly lower in the responder group compared with the non‐responder group (Figure 9E), suggesting these genes and the seven genes score may be involved in mediating resistance to immunotherapy.

## 4 Discussion

Hepatocellular carcinoma remains a formidable clinical challenge due to its high molecular heterogeneity, propensity for late‐stage diagnosis, and the frequent development of resistance to systemic therapies [2]. In this study, we addressed this challenge by integrating single‐cell transcriptomics with bulk multi‐omics data to develop and validate a novel prognostic signature rooted in the biology of the tumor microenvironment. Our central finding is the identification of a 7‐gene signature (*SLC41A3*, *DCAF13*, *PPM1G*, *NDC80*, *FAM83D*, *FUCA2*, *UQCRH*) derived from a subpopulation of macrophages exhibiting high intrinsic resistance to EGFR‐TKIs. This signature not only robustly stratifies HCC patients into distinct prognostic groups but also provides valuable insights into the mechanisms of therapeutic resistance and potential avenues for personalized intervention.

Our initial unbiased analysis of the HCC TME using scRNA‐seq data compellingly demonstrated that macrophages, above all other cell types, harbored the strongest gene expression signature associated with EGFR‐TKI resistance. This aligns with a growing body of evidence implicating TAMs as central players in mediating resistance to various cancer therapies, including targeted agents and chemotherapy [18]. TAMs can orchestrate a pro‐tumorigenic and drug‐resistant niche by promoting an immunosuppressive microenvironment, secreting growth factors, and remodeling the extracellular matrix, thereby shielding cancer cells from therapeutic assault [19, 20]. Our identification of a specific macrophage sub‐cluster (Macrophage_5) with the highest resistance score underscores the functional heterogeneity within the TAM compartment and highlights the importance of dissecting these subpopulations to uncover key resistance drivers.

The seven genes constituting our prognostic signature are functionally linked to core cancer hallmarks, including proliferation, cell cycle regulation, and metabolism. Several genes, including *NDC80*, *FAM83D*, *DCAF13*, and *PPM1G*, are directly involved in promoting cell cycle progression and inhibiting apoptosis. *NDC80*, a core component of the kinetochore complex, is crucial for mitosis, and its overexpression in HCC is linked to proliferation and poor outcomes [21, 22]. *FAM83D* is a potent oncogene that activates key signaling pathways like Wnt/β‐catenin and MEK/ERK to drive proliferation and invasion [23]. Similarly, *DCAF13* and *PPM1G* have been shown to promote HCC progression by degrading tumor suppressors or modulating oncogenic signaling [24, 25]. Together, these genes represent a coordinated module driving aggressive tumor biology.

The other signature genes point to metabolic and microenvironmental interactions. *SLC41A3*, a mitochondrial magnesium transporter, has been identified as an adverse prognostic biomarker in HCC whose expression is linked to immune cell infiltration [26, 27]. *FUCA2*, an α‐L‐fucosidase, has been reported to promote tumor cell invasion [28]. Intriguingly, our analysis linked its expression to broad sensitivity across numerous anti‐cancer agents, suggesting a complex role that warrants further investigation. The inclusion of *UQCRH*, a subunit of the mitochondrial complex III, in our adverse prognostic signature is also notable, as it has been described as a tumor suppressor in some contexts [29]. This apparent contradiction may reflect the context‐dependent function of metabolic genes in cancer; in the aggressive, high‐risk HCC subtype, its upregulation could be part of a compensatory metabolic stress response that ultimately fails to halt tumor progression. In two single‐cell immunotherapy datasets, lower expression of *UQCRH* and *PPM1G* was observed in responding patients, further implicating these genes in therapy resistance and highlighting their potential as therapeutic targets.

A major strength of our model is that the risk score was validated as an independent prognostic factor, outperforming traditional clinical parameters in multivariate analysis, and was successfully validated in the external GSE76427 cohort. This suggests that our biology‐driven signature captures essential molecular features of tumor aggressiveness not encapsulated by clinical staging alone. Furthermore, our multi‐omics investigation provided deeper mechanistic insights. The positive correlation between the genes' expression and their CNV levels suggests that genomic amplification is a key mechanism driving their upregulation in HCC, while the hypomethylation of genes like *SLC41A3* points to epigenetic dysregulation as another contributing factor.

The analysis of the risk score in the context of immunotherapy and targeted therapy yielded clinically actionable hypotheses. The finding that the low‐risk group is characterized by higher immune cell infiltration and a lower TIDE exclusion score provides a strong rationale for their favorable prognosis and suggests these patients are more likely to benefit from ICIs. Conversely, the high‐risk group's immune‐excluded phenotype suggests they may not respond well to single‐agent ICIs and might instead benefit from therapies designed to overcome immune exclusion, such as agents targeting TGF‐β or CXCR4 [30]. Furthermore, the predicted sensitivity of the high‐risk group to Sorafenib and Navitoclax opens avenues for tailored therapeutic strategies in this poor‐prognosis population.

The origin of our 7‐gene signature from an EGFR‐TKI‐resistant macrophage population provides a key insight. The molecular architecture of acquired resistance to EGFR‐TKIs in other cancers, such as NSCLC, is well‐characterized and highly relevant. When tumor cells develop resistance, they often activate other pathways such as RAS/RAF/MAPK [31] and Apoptotic Blockade (BCL‐2/MCL‐1) [32]. Sorafenib, as a RAF inhibitor, has been shown to be effective in EGFR‐TKI‐resistant NSCLC cell lines because it directly overcomes the RAS/RAF/MAPK‐driven resistance. Navitoclax, in turn, targets the BCL‐2 dependency [33].

To enhance the translational relevance of our findings, it is crucial to consider the practical steps for clinical application. A significant gap often exists between the publication of prognostic gene signatures and their implementation in routine clinical practice. This gap is frequently due to challenges in independent validation and the need for standardized, feasible detection platforms. A key translational step would be the development and analytical validation of a robust, multi‐gene RT‐qPCR assay for this 7‐gene signature. This platform is widely available in clinical pathology labs and would allow for the signature's subsequent clinical validation in large, independent patient cohorts. Once validated, this assay could be integrated with key clinical parameters (similar to the nomogram presented in Figure 4C), to create a practical, easy‐to‐use prognostic tool. Such a nomogram would provide a quantitative, personalized risk assessment, substantially increasing the clinical impact of our model by aiding in patient stratification and therapeutic decision‐making.

This study has several limitations. First, the biological functions of the seven signature genes and their collective role in mediating TKI resistance in macrophages need to be experimentally validated through in vitro and in vivo functional studies. For instance, co‐culture experiments using macrophages and HCC cell lines could be employed to functionally test whether the knockdown of these genes in macrophages can re‐sensitize HCC cells to TKIs. Second, while the scRNA‐seq data pointed to macrophages as the source of the signature, the model was built and validated using bulk TCGA data, which reflects an averaged signal from a mix of cell types. Future studies using spatial transcriptomics could help to clarify the precise cellular interactions and confirm the in situ expression of this signature within macrophage‐rich tumor regions.

In conclusion, we have developed and rigorously validated a novel 7‐gene signature derived from EGFR‐TKI resistant macrophages that effectively predicts prognosis in HCC. This signature provides a window into the molecular underpinnings of therapeutic resistance and offers a valuable tool for patient stratification. The associated genomic and therapeutic insights lay the groundwork for developing personalized treatment strategies to improve outcomes for patients with this deadly disease.

## Funding

This work was supported by the following funds: Natural Science Foundation of Shanxi Province (No. 202303021222129); Shanxi Provincial Health Commission (No. 2024048); Shanxi Medical University Doctoral Start‐up Fund Project (No. XD2245); Shanxi Medical University Provincial Doctoral Fund Project (No. SD2302); Shanxi Provincial Health Commission Scientific Research Project Plan (No. 2021149); Shanxi Bethune Hospital Scientific Research Project Plan Academy Level Scientific Research Fund (No. 2021YJ17); Natural Science Foundation of Shanxi Province (No. 202103021224361); Shanxi Bethune Hospital “136” Hospital‐level Open Fund (No. 2021YZ01); Fund programs: Shanxi Provincial Medical Association Physician Research Project (YSXH‐QL2024ZHZL016); Shanxi Cancer Hospital of Chinese Academy of Medical Sciences Shanxi Hospital Doctoral talents sailing fund (QH2023035); Doctoral Research Fund of Shanxi Cancer Hospital (2022BZJJ01).

## Conflicts of Interest

The authors declare no conflicts of interest.

## Supporting information


**Data S1:** Supporting Information.

## Data Availability

The datasets analyzed during the current study are available in the Gene Expression Omnibus (GEO) repository (scRNA‐Seq: GSE149614; bulk RNA‐Seq: GSE10186, GSE76427, GSE27150), the scICB database (immunotherapy scRNA‐Seq: GSE206325 and Liu_HCC), and The Cancer Genome Atlas (TCGA) portal (https://portal.gdc.cancer.gov/).
