# Supplementary material for: A Macrophage‐Derived 7‐Gene Signature Predicts Prognosis and Therapeutic Response in Hepatocellular Carcinoma
Source: IUBMB Life. 2025 Dec 7;77(12):e70079. doi: 10.1002/iub.70079 (PMC12683284; doi:10.1002/iub.70079)
Supplement: Supplementary file 1 — Data S1: Supporting Information. [file IUB-77-0-s001.pdf]

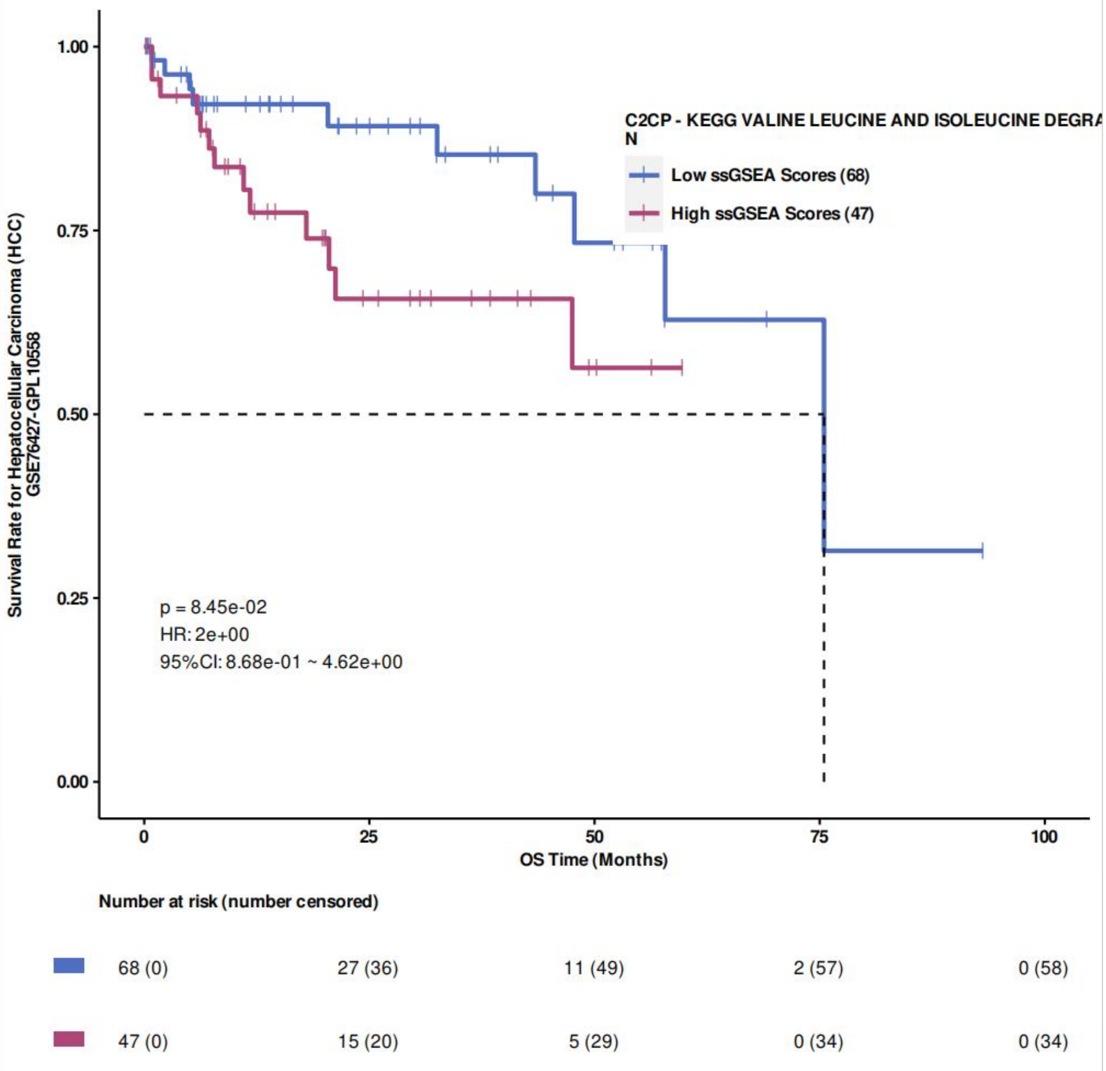

Figure S1 Survival curves with high and low scores of BCAA GSVA

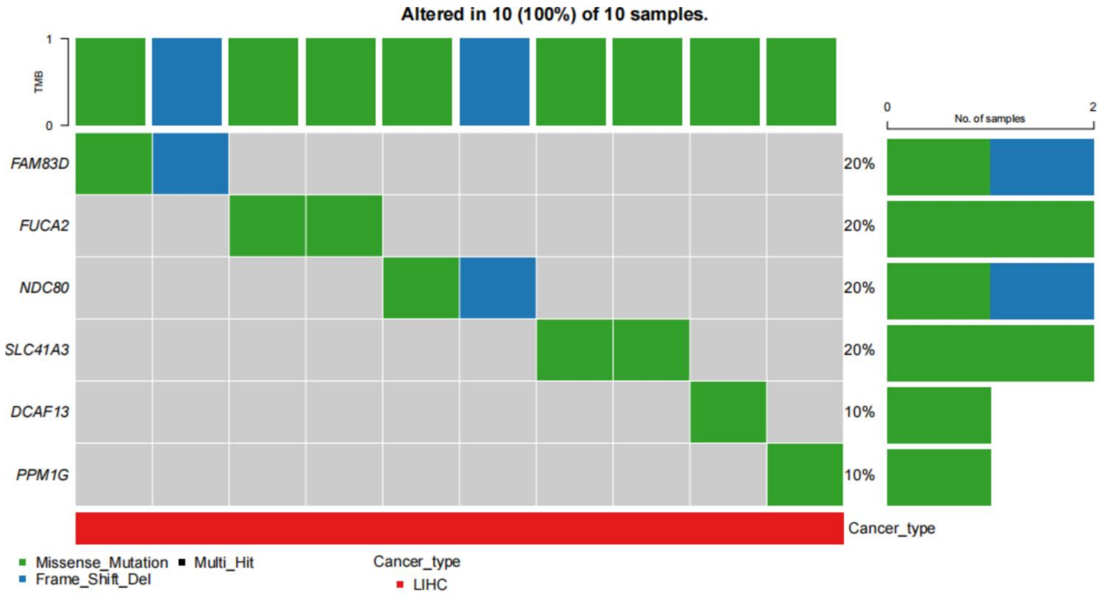

Figure S2 Distribution of mutation frequency of characteristic genes in TCGA-LIHC

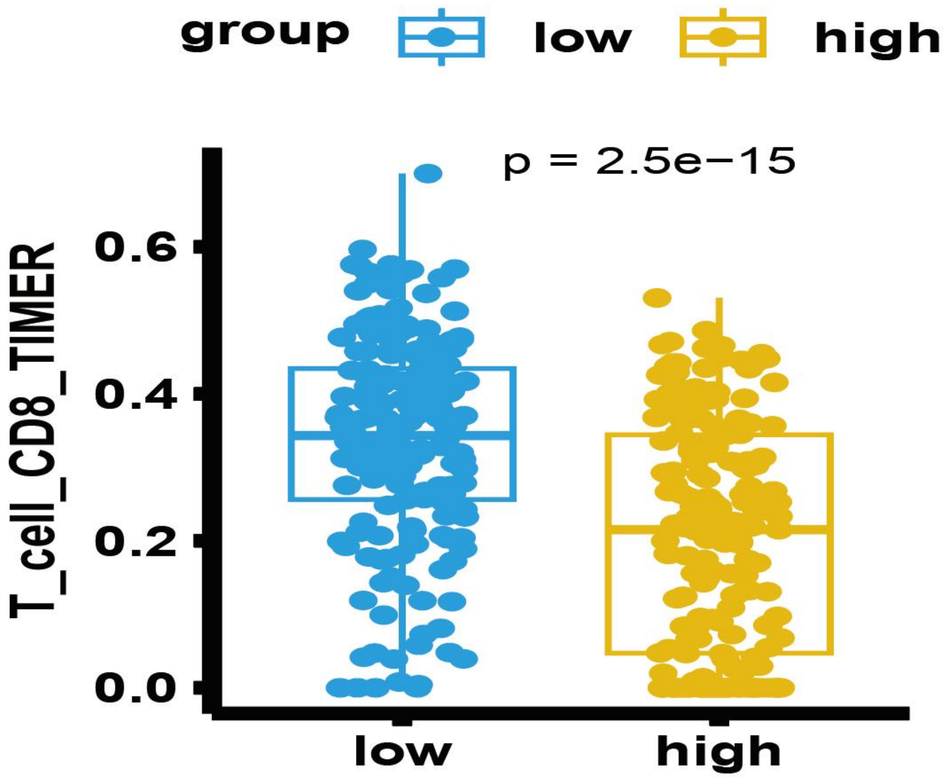

Figure S3 Distribution of CD8+ T cells in high and low groups

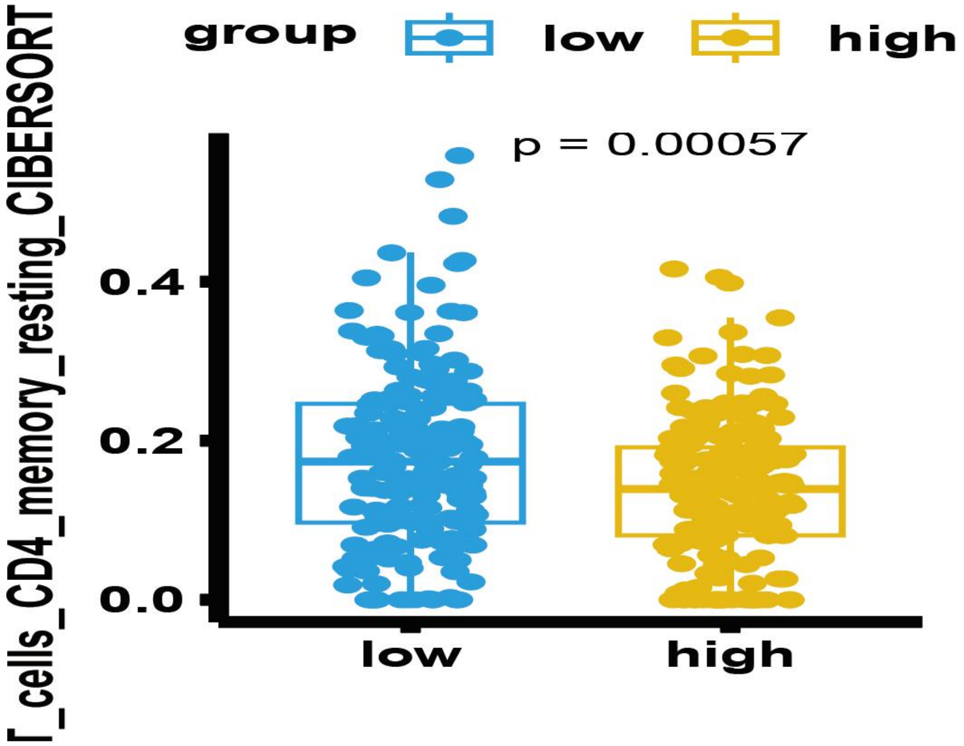

Figure S4 Distribution of CD4+ T cells in high and low groups
